# The Important Contribution of Non-*Saccharomyces* Yeasts to the Aroma Complexity of Wine: A Review

**DOI:** 10.3390/foods10010013

**Published:** 2020-12-23

**Authors:** Elliot Borren, Bin Tian

**Affiliations:** Faculty of Agriculture and Life Sciences, Lincoln University, Lincoln 7647, New Zealand; elliot.borren@lincolnuni.ac.nz

**Keywords:** aroma, fermentation, non-*Saccharomyces*, wine, yeast

## Abstract

Non-*Saccharomyces* yeast plays an important role in the initial stages of a wild ferment, as they are found in higher abundance in the vineyard than *Saccharomyces cerevisiae*. As such, there has been a focus in recent years to isolate these yeast species and characterize their effect on wine fermentation and subsequent aroma. This effect on wine aroma is often species and strain dependent, as the enzymatic profile of each yeast will determine which aroma compounds are formed as secondary metabolites. Semi-fermentative yeast, such as *Hanseniaspora* spp., *Candida* spp. and *Metschnikowia pulcherrima*, are commonly in high abundance in fresh grape must and have diverse enzymatic profiles, however they show a weak tolerance to ethanol, limiting their impact to the initial stages of fermentation. Fully fermentative non-*Saccharomyces* yeast, characterized by high ethanol tolerance, are often found at low abundance in fresh grape must, similar to *Saccharomyces cerevisiae*. Their ability to influence the aroma profile of wine remains high, however, due to their presence into the final stages of fermentation. Some fermentative yeasts also have unique oenological properties, such as *Lanchancea thermotolerans* and *Schizosaccharomyces pombe*, highlighting the potential of these yeast as inoculants for specific wine styles.

## 1. Introduction

For thousands of years, throughout the history of winemaking, fermentation occurred naturally without an understanding of how it worked and the microorganisms involved [1]. However, since Louis Pasteur discovered yeasts and their role in fermentation in 1866, this biochemical process has been a major area of research [2]. The fermentation of grape must to wine involves the conversion of grape sugars to ethanol and carbon dioxide through yeast metabolism. A wine ferment, however, is a demanding environment and yeasts performing fermentation are required to be tolerant to low pH, high levels of sugar and sulfur, low levels of oxygen, and also high ethanol concentrations as fermentation runs to completion. Of all the yeasts studied, the most attention has been given to *Saccharomyces cerevisiae*, as it is best adapted to survive in these harsh conditions of a wine ferment [3]. In fact, for the majority of untreated wine ferments, *S. cerevisiae* will be the single dominant yeast present at the end of fermentation [4]. As such, highly effective cultures of *S. cerevisiae* are available to buy as an inoculant for commercial wine ferments and *S. cerevisiae* is commonly referred to as simply “the wine yeast” [2].

While *S. cerevisiae* is found on harvested grapes and in wineries in low abundances [5], there is a greater diversity of non-*Saccharomyces* yeasts that are present on grapes and in grape must. As many of these yeasts are found in grape must at a far higher abundance than *S. cerevisiae,* they are in a much better position to begin fermentation [6]. For many winemakers, these non-*Saccharomyces* yeasts represent potential sources of wine spoilage as they are uncontrolled, have diverse and unknown fermentation kinetics, and can produce higher concentrations of off-aromas [4]. In conventional winemaking, to avoid the effect of the non-*Saccharomyces* yeasts, the grape must is quickly inoculated with a large culture of *S. cerevisiae* [7]. This allows the resulting ferment to have a much higher chance of avoiding any negative issues. Proponents of natural wine, however, believe this moves wine away from a natural product into an industrial one and by allowing the indigenous yeasts to perform fermentation you retain the traditional flavors and aromas of the wine [2]. This is due to two reasons: Firstly, non-*Saccharomyces* yeasts have been shown to produce a greater range of volatile metabolites during fermentation than *S. cerevisiae*. Secondly, yeast populations have been found to be regionally distinct, a concept coined microbial terroir [8]. The concept of terroir suggests that the environment in which the grapes are grown leaves an aromatic fingerprint on the wine that is unique, and it is suggested that this effect extends to the regional yeasts that perform the fermentation.

## 2. Volatile Compounds Released by Extracellular Enzymes of Yeast

The major difference between how wine yeasts produce aroma compounds during fermentation stems from the production of enzymes. Yeasts contain genes that encode enzymes that perform important roles in their survival [7]. Some of these enzymes are secreted outside the cell membrane to break down complex compounds and polymers in their surroundings. This provides energy and nutrients for the yeast, however, different yeasts produce different abundances of these enzymes. Some of these enzymes are directly responsible for the breakdown of sugar to ethanol and will therefore determine how rapidly fermentation will occur [7]. Other enzymes catalyze the formation of primary and secondary aroma compounds in wines. These are summarized in Table 1, along with a description of the aroma compound. Due to the diversity of indigenous non-*Saccharomyces* yeasts, there is a much wider range of extracellular enzymes produced during a wild fermentation than when inoculating with a monoculture of *S. cerevisiae* [2]. Therefore, it can be expected that a wider range of volatile compounds will be formed in a wild ferment. Modern gene sequencing techniques have also shown that some non-*Saccharomyces* yeasts encode for a greater amount of extracellular enzymes than *S. cerevisiae* [9]. As such, the isolation and characterization of these yeasts have been a major source of interest in the literature in recent years, intending to develop novel yeast starter cultures for commercial wine production.

## 3. Yeast Classification

Yeasts found in the vineyard can be split into three categories based on their potential to perform fermentation [10]. The first category is oxidative yeasts such as *Rhodotorula*, *Cryptococcus,* and *Aerobasidium* species. These are the most common yeast isolated on grape berries immediately after veraison when there is a growing sugar content in the grapes, but less competition from other yeasts [11]. However, in the middle and late stages of grape ripening, their abundance in relation to more fermentative yeast species tends to decline as there is more competition for nutrients. While they are still often present on grapes at harvest time, they are often ignored by winemakers as they do not play a role in the fermentation of the grape sugars [12]. This is not to say that they have no effect on wine aroma and two oxidative yeast species isolated from grape must, *Sporobolomyces roseus* and *Aureobasidium pullulans*, were shown to produce 10 volatile compounds typically found in red wine when left in a wine-like solution for 5 days [13]. While further research is required to quantify their influence on overall wine aroma, this study does suggest that oxidative yeast cannot be completely discounted when studying indigenous ferments.

The second category is oxidative-fermentative yeasts, otherwise named as semi-fermentative yeasts, some of the common genera being *Hanseniaspora*, *Candida,* and *Metschnikowia* [7]. These yeasts survive well on grape berries and are typically the most abundant yeasts found in grape musts and the early stage of fermentation. The fermentative ability of these yeasts is often low in wine like conditions, and many cannot tolerate the high ethanol concentrations of the later part of a ferment. However, this is very variable between species and some species such as *Hanseniaspora vineae* and *Candida zemplinina* (also known as *Starmellera bacillaris*) have been shown to survive in around 10% ethanol by volume in winemaking conditions [14]. The most commonly found yeast on mature grapes are *Hanseniaspora*, known also by its anamorph *Kloeckera* and in some cases as much as 60% of the yeast isolated from grape must are species of *Hanseniaspora* [15], often followed in abundance by several fermentative and non-fermentative species of *Candida*, *Metschnikowia* and *Pichia*.

The final category is highly fermentative yeasts of the genera *Saccharomyces*, *Torulaspora,* and *Lachancea*. Typically these yeasts are present on grapes in low concentrations unless there is damage to the grapes, either mechanical or from spoilage fungi and bacteria [10]. This allows for greater access to sugars and other nutrients from within the grape and occasionally in this situation, highly fermentative yeasts may dominate the microbiota of grapes and initial grape must [7]. These yeasts tend to be very tolerant towards alcohol, and have good fermentation rates in wine like conditions, meaning they tend to dominate in the later stages of fermentation. While *Saccharomyces* species are the most commonly associated with wine ferments, other species such as *Torulaspora delbrueckii* and *Lachancea thermotolerans* are commonly found and active in the later stages of indigenous ferments and have been isolated as commercially available inoculants [16].

## 4. Oenologically Important Non-*Saccharomyces* Yeasts

The emphasis of this review will be on semi- and highly fermentative yeasts found in the greatest abundance on fully ripe grapes, as well as select yeasts that have distinct oenological characteristics. A summary of these yeasts is shown in Table 2. These yeasts are likely to have the greatest impact on both indigenous wine ferments and as deliberately used starter cultures for commercial ferments, either alone or in combination with *S. cerevisiae*.

*Hanseniaspora* species represent the most abundant yeasts found in vineyards, giving them the greatest opportunity to influence the wine quality of a wild ferment [17]. As such, *Hanseniaspora* yeasts have been widely studied and in total ten species have been isolated, the most common two being *H. uvarum* and *H. guilliermondii* [18]. The abundance of *Hanseniaspora* was highlighted by an investigation into the regional abundances of microorganisms in California vineyards, which found that *H. uvarum* made up around 5% of the total microbiota, including all bacteria and other fungi [8].

*Hanseniaspora* species are semi-fermentative yeasts and tend to have a low ethanol tolerance of between 3–5% (*v*/*v*). Despite have slow fermentation kinetics, they have been found to consume a large amount of nitrogen at the beginning of fermentation, which can interrupt the growth of other yeast species, particularly *S. cerevisiae* [19]. Analyzing daily samples of a wild ferment with PCR found *H. uvarum* at high abundances until around day 3 of fermentation, at which point the abundance of *S. cerevisiae* was still less than 1% [20]. The interruption of the growth of highly fermentative yeasts can cause slow and sluggish ferments when high levels of *Hanseniaspora* species are present, often beginning partway through fermentation, once the ethanol level becomes too high for *Hanseniaspora* to tolerate [21]. For this reason, *Hanseniaspora* dominated ferments should be either co-inoculated or sequentially inoculated with fast-growing *S. cerevisiae* strains, and yeast nutrient levels maintained to ensure an environment where populations of other yeasts have a better chance to grow [19].

One of the major characteristics of *Hanseniaspora* species is to increase the concentration of acetate esters, which give the wine a positive fruity aroma, as well as higher alcohols and sulfur-containing compounds [39]. These attributes tend to lend themselves to certain wine styles, such as aromatic white wines, where these compounds already play an important role in their aroma profiles.

A study looking at the effect of inoculating grape must before cold maceration found *H. uvarum* produced the largest concentrations of isoamyl acetate and isobutyl acetate, contributing banana and strawberry aromas to wine [40]. Another study found that when co-fermented with *S. cerevisiae*, the presence of *H. uvarum* increased the concentration of acetate esters, especially isoamyl acetate, as well as some common short-chain ethyl esters [41]. These results have been confirmed on an industrial scale also, with no significant difference in final acetate esters concentration found in musts co-inoculated with *H. uvarum*/*S. cerevisiae*, when compared to lab and pilot ferments [22]. These studies all confirmed, however, that paired with the increase in acetate esters, comes the increased concentrations of other acetate compounds ethyl acetate and acetic acid [22,40,41]. These two compounds are the main contributors to volatile acidity, a common wine fault that gives the wine vinegar or ethereal aroma and at low concentrations are thought to increase the complexity of the aroma of indigenous ferment.

To further explore the effect of *H. uvarum* on wine aroma, studies have explored the difference between sequential and co-inoculation of *H. uvarum/S. cerevisiae* [23,42]. An increase in acetate ester concentration was observed with a sequential inoculation after 48 h, something not observed with co-inoculation studies, where *H. uvarum* was found to be a weak competitor with *S. cerevisiae*. When excessive *H. uvarum* yeast was used for the initial inoculation, a large increase in ethyl acetate and volatile phenols was observed, that provided a nail polish remover to the wine [42]. As a sequential inoculation would closer mimic an indigenous ferment, which would be initially dominated by *Hanseniaspora* species and other semi-fermentative yeasts, this increase in ethyl acetate likely to contribute to the rise in volatile acidity associated with these techniques. An investigation into the abundance of *Hanseniaspora* yeasts present in the vineyard could provide winegrowers useful insight into the efficacy of sequential inoculations and indigenous ferments.

*Hanseniaspora guilliermondii*, the second most abundant *Hanseniaspora* spp., have also been shown to have an increased concentration of acetate esters, such as 2-phenyl-ethyl acetate, associated with rose and honey aromas, as well as hexyl acetate and isoamyl acetate [3]. *H. guilliermondii* have been shown to produce large amounts of heavy sulfur containing compounds during fermentation also, such as 3-mercapto-1-propanol and trans-2-methyltetrahydrothiophen-3-ol, which have rancid or sweaty aromas [24]. *H. guilliermondii*, similar to *H. uvarum*, are well placed to affect the beginning of wild ferments due to their abundance at the beginning of fermentation and therefore it is likely that they contribute to the complexity of wild ferments, contributing both positive and negative aromas.

*Hanseniaspora vineae*, formally called *Hanseniaspora osmophila*, has also been well studied, despite not being particularly abundant in wild ferments [7]. It shows better fermentative abilities than other *Hanseniaspora* species, such as improved fermentation kinetics and greater ethanol tolerance of 10% by volume [14]. This allows *H. vineae* to continue fermenting sugars into the middle and later stages of the ferment, allowing it to have a greater overall effect on the aromatic profile of a wine [17]. A sensory study of Sauvignon Blanc wines co-fermented with *H. vineae* and *S. cerevisiae* found, similar to other *Hanseniaspora* species, an increase in the concentration of acetate esters, especially 2-phenylethyl acetate [43]. A sensory evaluation of the resulting wine showed an increase fruity characters when compared to wines inoculated with just *S. cerevisiae*, highlighting the potential for inoculants of *H. vineae* to be used in the production of young fruity wine styles. *H. vineae* has also been shown to produce lower levels of higher alcohols than other *Hanseniaspora* species, at similar levels to those produced by *S. cerevisiae* [44]. This study also interestingly found *H. vineae* produced ethyl guaiacol, commonly associated with the spoilage yeast *Brettanomyces*, at above its aromatic threshold, as well as other rare compounds not found with *S. cerevisiae*, such as N-acetyl tyramine and 1H-indole-3-ethanol acetate ester [44]. These compounds do not have well-established formation pathways and further research and as such it is not established how *H. vineae* produced these volatile compounds. As the ability to produce these unusual volatile compounds is likely to be strain specific, further isolation and characterization of novel strains of *H. vineae* may be required before hailing *H. vineae* a potential individual inoculant.

Of the ten species of *Hanseniaspora* isolated, six have been fully sequenced, *H. uvarum*, *H. guilliermondii*, *H. meyeri*, *H. optuntiae*, *H. clermontiae* and *H. vineae* [17]. The lack of fermentative ability may be able to be explained by *Hanseniaspora* species containing fewer genes coding for alcohol dehydrogenase enzymes compared to *S. cerevisiae* [45]. These enzymes are involved in the final step of the glycolytic pathway and have been found at higher levels in *H. vineae* compared to *H. uvarum* and *H. guilliermondii*, allowing for its greater tolerance for ethanol. When looking at their effect on wine aroma, the genes *IAH1* and *ATF2* have been shown to contribute towards acetate ester formation in *S. cerevisiae*, coding for esterase, and alcohol acetyltransferase enzymes. These genes have been found in all *Hanseniaspora* species at a greater rate than *S. cerevisiae* allowing them to produce high levels of acetate esters [45]. The genes for another alcohol acetyltransferase enzyme, *EHT1*, which contributes short-chained ethyl esters have been found only in the species *H. vineae*, which would contribute to the increase in fruity aromas observed in studies with this yeast [46].

*Candida* species are found in high abundances in the vineyard, although are typically unable to fix nitrogen or grow in an anaerobic environment, such as a wine ferment [47]. One notable exception to this is *C. zemplinina*, commonly referred to in the literature by its asexual anamorph *Starmerella bacillaris*. *C. zemplinina* was previously described as *C. stellata* until many strains were reclassified in 2011 as a separate species [6]. As such, many studies isolating strains of *C. stellata* found before 2011, may have indeed been strains of *C. zemplinina*. This has caused some confusion in the literature and may explain why the fermentation properties and aromatic profiles of *C. zemplinina* differ when measured in different studies. Certainly, even now *C. zemplinina* can be thought of as a heterogeneous yeast species, easily confused with other similar yeast species [47].

*C. zemplinina* has been found to be spread by fruit flies to mature ripening grapes [25], which may explain why it is commonly found in high abundance in the must of freshly pressed grapes. Because of its superior fermentative abilities and ethanol tolerance, > 10% (*v*/*v*), to many other non-*Saccharomyces* yeasts, it has been found to be the most abundant yeast in the middle of indigenous ferments and still present, although at lower abundances, at the end of fermentation [15]. This means that in indigenous ferments *C. zemplinina* will compete with *S. cerevisiae* for dominance once the rising ethanol concentration kills off other semi-fermentative yeast species. The competition between *C. zemplinina* and *S. cerevisiae* has been shown to be beneficial for both yeasts also, as *C. zemplinina* is a fructophilic yeast, meaning it shows a preference for fructose during fermentation, over glucose, the preferred sugar of *S. cerevisiae*. Because of this, when co-inoculated with *S. cerevisiae*, or inoculated sequentially, increased fermentation kinetics are observed with both yeasts consuming their preferred sugar [48].

*C. zemplinina* has been shown to produce a wide range of extracellular enzymes, such as pectinases, glycosidases, and glucanases, allowing it to significantly influence the aromatic profile of a wild ferment [47]. These enzymes will increase the primary fruit aromas of wine by cleaving terpenes and isoprenoids from their sugar-bound precursors. Populations of *C. zemplinina* do have a high level of genetic diversity in winemaking environments, however, so this may not be representative of the entire species [49].

*C. zemplinina* has been well reported to produce high levels of glycerol, which provides a sweet taste and can improve the mouthfeel of wine [2]. However, studies have not always been in agreement as to how the presence of *C. zemplinina* influences the volatile compounds or the aroma profile of the wine. Co-inoculation of Sauvignon Blanc must with *C. zemplinina*/*S. cerevisiae* has been reported to increase the concentrations of terpenes and lowered the concentrations of aldehydes, acetate esters, and higher alcohols compared to a pure culture of *S. cerevisiae* [50]. In comparison, a similar co-inoculation with Macabeo must increased the concentration of ethyl esters, short-chain fatty acids, and higher alcohols [26]. A third study found that despite having a higher concentration of esters, Chardonnay wines scored lower sensory scores when inoculated with *C. zemplinina* and *C. zemplinina*/*S. cerevisiae* then pure cultures of *S. cerevisiae* [51]. The study described *C. zemplinina* as providing a sauerkraut/ethyl acetate aroma, which competed with the fruity aromas and negatively affected the wine.

These differences in these studies may be related to heterogeneous nature of *C. zemplinina* and how it is commonly misclassified as other species of yeast. Alternatively, before the reclassification of *C. zemplinina*, *C. stellata* strains were found to produce a very complex enzyme profile, which was highly strain dependant [52]. Likely, many of these strains were indeed species of *C. zemplinina*, which means this could also be the reason for the confusion. Either way, further research sequencing novel strains of *C. zemplinina* and characterizing their effect on wine aroma is required before being confident in its use for commercial winemaking. It may also be that while *C. zemplinina* is well suited to the role of increasing aromatic complexity in indigenous ferments, isolating individual strains for use as pure inoculant cultures will not increase complexity in the same manner [2].

*Metschnikowia pulcherrima*, also known by its anamorph *Candida pulcherrima*, is another common semi-fermentative yeast in indigenous ferments, commonly found on grapes and other organic tissues like flowers, fruit, and tree sap [16]. It has been found to be abundant in grape must at levels up to 39% of the yeast populations [53] although commonly is found at abundances between 5 and 20% [39]. *M. pulcherrima* has a relatively low fermentative ability, compared to other non-*Saccharomyces* yeast species, with a slow depletion of nitrogen observed and less CO_2_ produced during fermentation [29]. Most strains also survive only until around 4% ethanol concentrations, limiting its role in indigenous ferments [54]. Despite this, *M. pulcherrima* has oenological properties that make it attractive to winemakers [55], and cultures are available to buy as a commercially available inoculant.

*M. pulcherrima* produces a pulcherrimin, a red insoluble pigment which acts as an antimicrobial compound towards other non-*Saccharomyces* yeasts [16]. A precursor to pulcherrimin, pulcherriminic acid, binds to iron(III) ions, which precipitate out of solution and thus stopping the growth of yeast which require iron for development, such as *Brettanomyces*, *Hanseniaspora*, *Candida, Pichia* [55]. It also is effective at preventing the growth and impact of spoilage fungi, both in must and on pre-harvest grapes, such as *Botrytis cinerea*, *Penicillium,* and *Alternaria* [56]. *Saccharomyces cerevisiae* tends not to be affected by pulcherrimin, making it a good fermentation partner for either a sequential or co-inoculation with *M. pulcherrima* [55]. *M. pulcherrima* also shows high proteolytic activity, rapidly breaking down proteins into amino acids, which can be used as a source of nutrients by *S. cerevisiae*, as well as being the substrates required in the formation of ethyl esters, along with ethanol [57].

A significant change in the aromatic profile of wines inoculated with *M. pulcherrima* has been reported, owing largely to an intense extracellular enzymatic activity [2]. This includes high β-glucosidase activity, which allows for the cleaving of free terpenes and thiols, providing a floral aroma to *M. pulcherrima* ferments and enhancing the varietal fruit aroma in many grape varieties [30]. Due to the increase in amino acids, a high concentration of ethyl esters is also common with *M. pulcherrima*, especially ethyl octanoate, although some reports suggest this may be strain specific [31]. This is because it is likely that variable concentrations of esterase enzymes exist between strains to catalyze this formation. A trained sensory panel confirmed these findings, as three aromatic white varieties, Sauvignon Blanc, Muscat d’Alexandria and Chenin Blanc obtained higher sensory scores when sequentially inoculated with *M. pulcherrima* and *Saccharomyces cerevisiae* cultures compared to a pure *S. cerevisiae* culture [58]. A more intense fruit flavor was reported for these wines, owing largely to an increase in terpenes such as limonene and ɑ-terpineol, and a variety of esters. The timing of the sequential inoculation has been found to be important however as if left to ferment alone for too long, *M. pulcherrima* has also been found to produce large amounts of ethyl acetate, negatively influencing wine aroma [59]. Further research to find the ideal timing and conditions around inoculation with *M. pulcherrima* would help winemakers further realize the potential of this yeast as a commercial inoculant, as well as understand its role in the initial stages of an indigenous ferment.

*Wickerhamomyces anomalus* is another yeast frequently isolated from grapes and must, which is also known as *Pichia anomala*, traditionally known as a film-forming spoilage yeast in bulk wines [32]. It has been found to tolerate low-pH wine and is quite resistant to ethanol, with some strains found to tolerate up to 12.5% by volume. Oenological interest in *W. anomalus* exists as they have been shown to produce a very high level of extra-cellular enzymes, such as β-glucosidases and β-D-xylosidases, in wine-like conditions [60]. As such, a high level of monoterpenes has been observed in Muscat grape juice treated with *W. anomalus*, as well as a high level of fruity acetate esters, such as 2-phenylethylacetate [61]. However, this propensity to produce acetate esters extends towards the production of ethyl acetate, which gives off an unpleasant solvent-like aroma in wine above concentrations of 150 mg/L. Even in co-inoculation studies with *S. cerevisiae*, most isolated cultures of *W. anomalus* tended to produce around 200 mg/L of ethyl acetate [33]. This severely impacts the use of *W. anomalus* as a fermentation yeast, although it must be noted, that wines produced in larger tanks (>100 L) contained lower concentrations of ethyl acetate, probably due to a more anaerobic environment [33]. This indicates that larger commercial ferments with *W. anomalus* may not suffer from the problem of increased ethyl acetate production, however, further research is required to confirm this theory.

*Lanchancea thermotolerans*, until recently known as *Kluyveromyces thermoteolerans*, is a semi-fermentative yeast typically found in low abundances in grapes, soil, and other vine organs [62]. It has been found in indigenous ferments, although typically only at low abundances in the middle stages of fermentation, often limiting the role it plays [53]. Certain terroir has been found to support the growth of *L. thermotolerans* however, and differences in the abundance of *L. thermotolerans* have been observed across Californian wine regions [27]. They have also been shown to play a role in creating the regionally specific aromas in certain Portuguese wine regions, highlighting the importance of *L. thermotolerans* to these wine regions and styles [63].

*L. thermotolerans* has been shown to have good oenological properties, with an ethanol tolerance of around 9% and a good fermentative ability, even when fermentation is dominated by *S. cerevisiae* [11]. It has been widely studied due to its ability to acidify wine ferments and commercially produced cultures are available to buy to be used in mixed fermentations with *S. cerevisiae*. *L. thermotolerans* achieves this by producing high concentrations of lactic acid, especially at the beginning of fermentation, which can drop the pH of wines at initial pH levels of 3.8–4 by more than 0.5 pH units [64]. By lowering the pH of the wine, the level of molecular SO_2_ increases at lower levels of total SO_2_, which protects the wine from spoilage yeasts and bacteria, such as *Brettanomyces*, over aging [62].

*L. thermotolerans* tends to produce acetic acid at low concentrations, between 0.3–0.5 g/L [54], well below its sensory threshold of 0.74 g/L [28]. Production of high levels of acetic acid, the main contributor to volatile acidity, is common with non-*Saccharomyces* yeasts and often is a hurdle when attempting to fully ferment wine in the absence of *Saccharomyces* species. Along with the ability of *L. thermotolerans* to acidify ferments, lowering volatile acidity has been a major reason behind its use commercially. This also allows *L. thermotolerans* to be used in combination with other highly fermentative non-*Saccharomyces* yeasts prone to acetic acid production, such as *Schizosaccharomyces pombe*. In fact, one study produced dry faultless wines, with low levels of acetic acid and ethyl acetate, when co-fermented with mixed *S. pombe*/*L. thermotolerans* cultures, something not achieved with pure cultures of *S. pombe* or mixed cultures of *S. pombe*/*S. cerevisiae* [34].

*L. thermotolerans* has been found to produce a number of compounds that positively influence wine aroma and flavour. Similar to some other non-*Saccharomyces* yeasts, such as *Candida zemplinina*, *L. thermotolerans* produces a high concentration of glycerol, the concentration of which increases with fermentation temperature and availability of oxygen [65]. There is an advantage with using *L. thermotolerans* when seeking to increase glycerol concentration in wine, however, as it is not accompanied by an increase in acetic acid. A high concentration of β-glucosidases and carbon sulfur lyases are also observed in ferments with *L. thermotolerans* [4]. This has been shown to increase select monoterpene concentrations, such as nerol and terpinen-4-ol and varietal sulfur compounds in Sauvignon Blanc and Syrah musts [66]. Both these varieties have been described as much more distinct when sequentially inoculated with *L. thermotolerans* and *S. cerevisiae*, compared to must inoculated with only *S. cerevisiae* [67]. The Syrah wines had an increase in 1-ethyl-1h-pyrrole-2-carboxaldehyde which provides a beneficial spicy, smoky aroma, whereas Sauvignon Blanc musts had significant concentrations of 4-methyl-4-sulfanylpentan-2-one (box-tree aroma) and 3-sulfanyl hexan-1-ol (grapefruit and passionfruit aroma) [67]. While these initial results are promising, it is hard to compare the results of different studies as fermentation conditions tend to vary wildly [64]. Further research exploring the ideal conditions for inoculations with *L. thermotolerans* would allow for a better description of its impact on wine aroma.

*Torulaspora delbrueckii* were the first non-*Saccharomyces* yeast suggested for commercial wine ferments, as they showed similar fermentative characteristics to *S. cerevisiae* [35]. A problem arose on a commercial scale as wineries tended to be contaminated with existing *S. cerevisiae* strains and *T. delbrueckii* gets overtaken and dominated by the presence of *S. cerevisiae* in wine ferments, even in small abundances [68]. Since then, some killer strains of *T. delbrueckii* have since been isolated and shown to suppress the growth of *S. cerevisiae* and other non-*Saccharomyces* yeasts by producing an antimicrobial toxin (Kbarr-1) encoded in an RNA virus (ScV-Mbarr-1) [69]. When allowed to dominate, these killer strains have been found to increase the complexity of wine aroma, by reducing the concentrations of common ethyl esters and increasing the concentration of lactones and lesser-known esters [70]. This reduces the fresh fruity aroma of the wines, replacing it with dried fruit/pastry aromas. Another factor with *T. delbrueckii* is that it tends to perform poorly when no oxygen is present. As such is more suited to red wine, because some oxygen is added to the ferment when breaking up the skin cap, compared to the strictly anaerobic conditions of white and sparkling ferments [71].

*Schizosaccharomyces pombe* is of oenological interest due to some unusual fermentation characteristics, despite not typically being associated with the grapes or must [72]. It is tolerant of low pH, high levels of SO_2_, and ethanol levels of 10–15% (*v*/*v*), depending on the strain [73]. While nitrogen requirements for *S. pombe* are much less than *S. cerevisiae*, it has a much slower growth rate, meaning ferments with pure cultures tend to be sluggish. The slow fermentation speed is made up for, however, as *S. pombe* has the ability to perform maloalcohlic fermentation, whereby along with sugar, malic acid is converted into ethanol and CO_2_, reducing the need for malolactic fermentation [74]. While *S. pombe* is a poor producer of extra-cellular enzymes and tends to provide the wine with increased volatile acidity and a muted fruit aroma, there are areas where *S. pombe* may excel. As previously mentioned in this essay, *S. pombe* has been used in a co-fermentation with *L. thermotolerans* to produce wine with low levels of acetic acid, no malic acid, and higher levels of total esters than commercial *S. cerevisiae* strains [34]. It has also been suggested as yeast for the secondary fermentation of sparkling wines, as it has a high release rate of polysaccharides during aging on lees [75]. In one study looking at using *S. pombe* for the secondary fermentation, both red and white sparkling wines were rated as high-quality and no difference in the taste was observed when compared with wines fermented with *S. cerevisiae* [76]. While the buttery and yeasty aromas tended to increase when fermented with *S. pombe* and the fruity and floral aromas of the white sparkling wines were partially lost. It has also been suggested as yeast to decrease the acidity of ice-wines and other dessert wines, where due to the low pH values and high residual sugar, lactic acid bacteria are unable to consume the malic acid [72].

*Pichia kluyveri* has been isolated in low abundance in grape and must samples [36,77], although relatively at much higher abundances in damaged grapes [36]. *P. kluyveri* is weakly fermentative, with an ethanol tolerance of around 4–5% (*v*/*v*) with a slow fermentation rate, similar to that of *M. pulcherrima* [78]. In Sauvignon Blanc must, it has been found to produce high concentrations of thiols, especially 3-mercaptohexyl acetate, when sequentially inoculated with *S. cerevisiae* [37]. This has led to the development of a *P. kluyveri* commercially available inoculant, which is described as the most potent thiol producing non-*Saccharomyces* inoculant available. *P. kluyveri*/*S. cerevisiae* sequential inoculations have been found to produce high concentrations of glycerol in Riesling must, similar to those produced by *L. thermotolerans*/*S. cerevisiae* [78], as well as an overall increase in ester and higher alcohol concentrations when compared to single inoculations of *S. cerevisiae* [38]. The compounds H_2_S and valeric acid, which confer rotten egg and rancid aromas respectively, were also found at increased concentrations in ferments containing *P. kluyveri*, although the origins of these aromas were not established [38].

## 5. Conclusions

There is much more to wine fermentation than the conversion of grape sugars to ethanol. The development of primary and secondary aromas in wine is a key role of yeasts during fermentation and research suggests that non-*Saccharomyces* yeasts produce a wider range of volatile aroma compounds than *S. cerevisiae*. This is because each species of yeast encodes for different concentrations of extracellular enzymes and these will have distinct effects on the aroma profile of a wine. There has been a plethora of research in recent years to describe how different non-*Saccharomyces* yeast will undergo wine fermentation and a number of these yeasts are now available to be purchased commercially. These investigations shed light on the fermentation kinetics of oenologically important non-*Saccharomyces* yeasts and how they will influence the aroma of the wine. This allows for greater management of both indigenous ferments and aids in the selection of commercially available inoculants to achieve the desired fermentation aromas. Many of these yeast characteristics are species and strain dependent and as such, isolating and characterizing novel species and strains is an ongoing aim of wine yeast research. Development of new commercially available inoculants will provide winemakers with further tools with which to develop styles and produce aromatically distinct wines.

## Figures and Tables

**Table 1:** Common volatile compounds produced during fermentation complete with the enzymes responsible, initial substrates, and their effect on the aroma.

Volatile Compounds	Enzyme Responsible	Substrates	Aroma Descriptors
Esters	Esterase, alcohol acetyltransferase	Alcohol + acid	Fruity, floral
Terpenes	Glycosidase	Terpenoid glycosides	Floral, varietal
Higher alcohols	Alcohol dehydrogenase	Amino acids	Low: Fruity High: Ethereal
Volatile phenols	Phenol reductase, decarboxylase	Carboxylic acids	Low: Smokey, bacon High: Barnyard, sweaty
Sulphur containing compounds	Sulphur lyase, alcohol dehydrogenase	Amino acids, thiols, natural sulphur	Sulphite: Rotten eggs Thiol: Tropical fruity
Volatile fatty acids	Decarboxylase, fatty acid sythase	Acetyl-CoA, malonyl-CoA	Vinegar, rancid, pungent

**Table 2:** Non-*Saccharomyces* yeasts with great oenological interests.

Yeast	Abundance on Grapes	Fermentation Rate	Ethanol Tolerance	Influence on Wine Aroma	References
*Hanseniaspora* spp.	High	Low	3–5%	Increased concentration of acetate esters, volatile acidity and higher alcohols. Overall increased aromatic complexity.	[16,20,21]
*Candida zemplinina*	High	Medium	10%	Increased concentration of ethyl acetate, terpenes and glycerol. Ester results mixed. Overall increased aromatic complexity.	[22,23,24]
*Metschnikowia pulcherrima*	Medium	Low	4–5%	Increased concentration of ethyl esters, terpenes and higher alcohols. Reduced volatile acidity.	[25,26]
*Wickerhamomyces anomalus*	Medium	Medium	12%	Increased concentration of acetate esters, monoterpenes. Very high ethyl acetate producer.	[27,28]
*Lanchancea thermotolerans*	Low	High	9%	Increased concentration of terpenes, lactic acid, and volatile sulphur compounds.	[29,30,31]
*Torulaspora delbruekii*	Low	High	14%	Substitute common fruity esters for lactones and uncommon esters.	[32,33]
*Schizosaccharomyces pombe*	Very low	Low	10–15%	Performs malo-alcoholic fermentation, increases acetic acid concentration and lowers overall fruity aromas.	[34,35]
*Pichia kluyveri*	Low	Low	4–5%	Powerful thiol producer, increased concentration of esters, higher alcohols and glycerol	[36,37,38]
